# Efficient Expression of Functional (α6β2)_2_β3 AChRs in Xenopus Oocytes from Free Subunits Using Slightly Modified α6 Subunits

**DOI:** 10.1371/journal.pone.0103244

**Published:** 2014-07-28

**Authors:** Carson Kai-Kwong Ley, Alexander Kuryatov, Jingyi Wang, Jon Martin Lindstrom

**Affiliations:** Department of Neuroscience, Perelman School of Medicine at the University of Pennsylvania, Philadelphia, Pennsylvania, United States of America; University of Michigan, United States of America

## Abstract

Human (α6β2)(α4β2)β3 nicotinic acetylcholine receptors (AChRs) are essential for addiction to nicotine and a target for drug development for smoking cessation. Expressing this complex AChR is difficult, but has been achieved using subunit concatamers. In order to determine what limits expression of α6* AChRs and to efficiently express α6* AChRs using free subunits, we investigated expression of the simpler (α6β2)_2_β3 AChR. The concatameric form of this AChR assembles well, but is transported to the cell surface inefficiently. Various chimeras of α6 with the closely related α3 subunit increased expression efficiency with free subunits and produced pharmacologically equivalent functional AChRs. A chimera in which the large cytoplasmic domain of α6 was replaced with that of α3 increased assembly with β2 subunits and transport of AChRs to the oocyte surface. Another chimera replacing the unique methionine 211 of α6 with leucine found at this position in transmembrane domain 1 of α3 and other α subunits increased assembly of mature subunits containing β3 subunits within oocytes. Combining both α3 sequences in an α6 chimera increased expression of functional (α6β2)_2_β3 AChRs to 12-fold more than with concatamers. This is pragmatically useful, and provides insights on features of α6 subunit structure that limit its expression in transfected cells.

## Introduction

Human α6β2β3* nicotinic acetylcholine receptors (AChRs) on dopaminergic neurons are important targets for drugs to treat nicotine addiction and Parkinson’s disease [1]–[5]. These AChRs are critical brain subtypes in processes involving movement, memory, reward, and learning [6], [7]. Self-administration of nicotine is inhibited by knockout of α6, β2, or α4 subunits, implying that (α6β2)(α4β2)β3 AChRs are necessary for presynaptically modulating dopamine release associated with reward and induction of nicotine-dependence [8]. Antagonists of α6β2β3* AChRs inhibit nicotine self-administration by reducing nicotine-induced release of dopamine [2]. Smoking-relevant concentrations of nicotine sustain smoldering activation of dopaminergic neurons through (α6β2)(α4β2)β3 AChRs [5], [9], [10]. α6β2β3* AChRs are more abundant in dopaminergic neurons in primates than in rodents, thus probably even more important in humans than in rodents [11]. Therefore, it is critical to be able to express functional human α6β2β3* AChRs to develop better drugs.

However, efficiently expressing the complex (α6β2)(α4β2)β3 subtype or the simpler (α6β2)_2_β3 subtype has been difficult [12]–[15]. Mixtures of free α6 and β2 subunits efficiently assemble (α6β2) binding sites, but not mature AChRs [12]. β3 subunits promote expression and nicotine-induced upregulation of human α6* AChRs expressed in transfected cell lines, but the amount of AChRs expressed is too small for detecting AChR function [13]. Use of subunit concatamers enabled expression in *Xenopus* oocytes of both human α6β2β3* AChR subtypes [16]. Linking subunits in pentameric concatamers results in efficient assembly of α6β2β3* AChRs [16]. A pentameric concatamer of (α6β2)(α4β2)β3 AChRs is efficiently transported to the surface [16]. However, transport of concatameric (α6β2)_2_β3 AChRs to the surface membrane is inefficient. It is important to determine what limits expression of these subtypes and to express them efficiently.

Chimeras reveal α6 sequences that limit expression of transfected α6* AChRs. Chimeras with the extracellular domain of α6 and the remainder of α3 or α4 expressed in combination with β2 are efficiently transported to the surface [12]. The large cytoplasmic domain of some AChR subunits promotes assembly and transport of AChRs [17]. This suggests that the large cytoplasmic domain of α3 or α4 might provide efficient transport to the surface that α6 does not. The cytoplasmic domain of α3 is smaller than that of α4 and more closely resembles the sequence of α6.

Analysis of chimeras of α6 and α3 identified α3 sequences that permitted expression of α6* AChRs and sequences of α6 that inhibited expression of α3 AChRs [16]. A region of α6 in the first half of transmembrane domain 1 inhibited expression of α3 AChRs [16]. This region contains a unique methionine at position 211 which is occupied by leucine in α3 and other α subunits. M211 is in a sequence that in α1 subunits governs stability, assembly, and transport to the cell surface [18]. This sequence is on the side of the α1 subunit that assembles with the accessory subunit (e.g. β1 in α1* AChRs or β3 in α6* AChRS) [19]. This suggests that the α6 sequence containing M211 might be important for associating with β3 accessory subunits. α6β2β3* AChRs are usually expressed in aminergic neurons at presynaptic locations [1], [20], [21]. These neurons may express a chaperone for assembly of β3 subunits with α6 that is missing in other cell types such as *Xenopus* oocytes or human embryonic kidney (HEK) cell lines. These cells efficiently assemble β3 with other α subunits to form AChRs such as (α4β2)_2_β3 [22].

Here we report efficient expression of (α6β2)_2_β3 AChRs in *Xenopus* oocytes using free subunits with only small changes in α6 subunits, while not altering AChR pharmacology or channel structure. We explored the effects of incorporating M211L and α3 cytoplasmic domain alone, and together, into chimeras with α6. M211L increased assembly with β3, and α3 cytoplasmic domain increased assembly with β2 and transport to the surface. Together, these two modifications synergistically permitted expression of high levels of (α6β2)_2_β3 AChRs from free subunits. These AChRs exhibited the same pharmacological properties as concatameric AChRs, but were expressed on the oocyte surface in much greater amounts.

## Materials and Methods

### Ethics Statement

The anesthesia (1 g/1 L Tricane) used and treatment of *Xenopus laevis* frogs in our research experiments was performed in accordance and under the strict guidelines of our approved protocol [Protocol #: 804234; Title: Studies Using Purified Acetylcholine Receptors; Renewed: Dec 17, 2013] by the IACUC Protocol Administration of the University of Pennsylvania, located on Suite 301S, 3624 Market Street, Science Center, Philadelphia, PA 19104, USA (Phone: 215-573-2540; Fax: 215-573-9438).

### Construction of α6/α3 Chimeras

We prepared chimeras from α3 and α6 subunits [12]. To replace the cytoplasmic domain of α6 with the corresponding part of α3, the *ApaL*I restriction site was introduced at position Ile297H is 298 of α6 cDNA using a QuikChange site-directed mutagenesis kit (Stratagene, La Jolla, CA). This restriction site is present in native α3 cDNA. Using this restriction site and the *Nco*I site that is common between α6 and α3 in the beginning of the M4 domain, we replaced the cytoplasmic domain of α6 with the α3 domain, leaving intact the M4 transmembrane domain and C-tail of α6. To form the α6_α3cyt-C_ construct, the cytoplasmic domain, M4 transmembrane domain and C-tail of α6 were replaced with α3 using an introduced *ApaL*I site and an *EcoR*I site from the psp64 (polyA) plasmid.

To make M211L chimeras, we made the M211L change in α6 sequence using a GAAGATTGCCGCTGTTTTACACG oligo and a QuikChange site-directed mutagenesis kit (Stratagene, La Jolla, CA) for native α6 or α6 with an α3 cytoplasmic domain.

### Concatameric Linker Construction

The pentameric concatamer β3−α6−β2−α6−β2 was made as described previously [16]. This used (Alanine, Glycine, Serine)_n_ ((AGS)_n_) linkers.

β3 and α6 joined with a Q_4_A_3_PAQ_3_AQA_3_PA_2_Q_5_ (QAP) linker to form the β3−α6 concatamer was used here. Using previously prepared β3 with a *BspE*I site at the end of the coding domain and α6 with a *Fsp*I site at the end of signal peptide [16], we inserted QAP linker with these oligos: CCGGGAACAGCAACAGCAAGCAGCGGCTCCGGCCCAACAGC.

AAGCACAGGCGGCTGCCCCCGCAGCGCAACAACAGCAACAGTGC and GCACTGTTGCTGTTGTTGCGCTGCGGGGGCAGCCGCCTGTGCTTGCTGTTGGGCCGGAGCCGCTGCTTGCTGTTGCTGTTC. This QAP linker is modeled on one used to express concatameric GABA_A_ receptors in HEK 293 cells [23]. To prepare the β3−α6 concatamer with the M211L chimera and α3 cytoplasmic domain, we used the unique site of *EcoR*V in the α6 sequence and the unique site of *Pvu*I in the psp64 (polyA) plasmid.

### DNA Preparation

Two microliters of DNA ligations were transformed into XL-10 Gold Ultracompetent cells (Stratagene, La Jolla, CA) using the protocol in the kit. Colonies were selected using the QIAquick spin miniprep kit. Miniprep DNA was tested for correct sequence by restriction enzyme digestion and subsequent agarose gel electrophoresis for correct size of fragments. DNAs were purified using Qiagen plasmid midiprep kit (QIAGEN) and concentrations were calculated using spectrophotometry.

### Oocyte Injection

*Xenopus laevis* oocyte harvest from *Xenopus laevis* frogs was performed in accordance with our approved IACUC protocol. The cRNA encoding desired subunits was synthesized from 1 µg of linearized cDNA templates in the pSP64 vectors using SP6 RNA polymerase from the mMessage mMachine kit (Ambion Inc, Austin, TX). Subunit cRNAs were mixed at a 1∶1∶1 ratio of α6:β2:β3 for constructs of free subunits. Dimeric concatamer was mixed with α6 chimera and β2 subunits at a 1∶1∶1 ratio. *Xenopus laevis* oocytes were injected with 100 ng of cRNA mix per oocyte, then incubated in 50% L-15 (Invitrogen, San Diego, CA), 10 mM HEPES, pH 7.5, 10 units/ml penicillin, 10 µg/ml streptomycin, and 50 µg/ml gentamycin [24]. This medium was refreshed daily, and replaced with gentamycin-free medium the day before recording.

### Surface Expression of AChR

Surface expression was determined by binding of ^125^I monoclonal antibody (mAb) 295 performed on the same day as electrophysiological recording of responses to 30 µM ACh. Groups of 8 oocytes were placed in an Eppendorf tube with 525 µl of L-15 media containing 10% horse serum and 5 nM ^125^I mAb 295 [25] at room temperature for 3 hours. Unbound ^125^I mAb was removed by 3 washes with 1 ml of L-15, then ^125^I bound to individual oocytes was determined in a γ-counter. Nonspecific binding was determined using non-injected oocytes.

### ^3^H Epibatidine Binding

Groups of 8 oocytes were homogenized in 1 ml of buffer A (50 mM NaCl, 50 mM sodium phosphate buffer, pH 7.5, 5 mM EDTA, 5 mM EGTA, 5 mM benzamide, 5 mM iodoacetamide, 2 mM phenylmethylsulfonyl fluoride). Then a crude membrane fraction was pelleted by centrifugation for 15 minutes at 13,400 rpm. Membrane proteins were resuspended by pipetting and solubilized in 150 µl of buffer A containing 2% Triton X-100 for 1 hour at room temperature. Debris was removed by centrifugation at 13,400 g for 15 min. Next, mAb 295 coated wells were loaded with aliquots of detergent extracts with 2 nM ^3^H epibatidine (PerkinElmer Life Sciences, Emeryville, CA) in a total volume of 100 µL in phosphate-buffered saline (PBS) buffer containing 0.5% Triton X-100 and 10 mM NaN_3_ [12]. These plates were left overnight on a shaker at 4°C. Wells were then washed three times with 0.5% Triton X-100 in PBS. ^3^H epibatidine bound to AChRs bound to the wells through mAb 295 or mAb 210 was eluted with 30 µl of 0.1 M NaOH and quantitated by liquid scintillation counting [22]. Nonspecific binding was determined using non-injected oocytes.

### Sucrose Gradient Sedimentation

Triton X-100-solubilized AChRs from oocytes were prepared as described above from groups of 30–50 oocytes [16]. Aliquots (150 µl) of the extracts, mixed with 2 µl of 1 µg/ml of *Torpedo californica* electric organ AChR, were loaded onto 11.3 ml sucrose gradients [linear 5–20% sucrose (w/v) in 10 mM sodium phosphate buffer, pH 7.5, that contained 100 mM NaCl, 1 mM NaN_3_, 5 mM EDTA, 5 mM EGTA, and 0.5% Triton X-100] [16], [26]. Gradients were centrifuged for 16 hours at 40,000 rpm in a SW-41 rotor (Beckman Coulter, Fullerton, CA) at 4°C. Fractions were collected at 15 drops per well from the bottom of the tubes. Fifty microliters of each fraction were transferred to mAb 295-coated wells to isolate β2-containing AChRs for measurement of ^3^H epibatidine binding, and 20 µl of each fraction were transferred to mAb 210-coated wells to isolate *Torpedo californica* AChRs used as internal molecular weight standards [26]. Fractions in mAb 295-coated wells were incubated with 2 nM ^3^H epibatidine at 4°C overnight. Fractions in mAb 210 coated wells was incubated with 1 nM ^125^I α bungarotoxin at 4°C overnight. Wells were then washed three times with PBS and 0.5% Triton X-100. Bound ^3^H epibatidine was determined by liquid scintillation counting.

### Electrophysiology

Electrophysiological recording was performed with a two-microelectrode voltage clamp amplifier (Oocyte Clamp OC-725; Warner Instruments, Hamden, CT) on *Xenopus* oocytes with a constant flow of ND-96 solution (96 mM NaCl, 1.8 mM CaCl_2_, 1 mM µgCl_2_, and 5 mM HEPES, pH 7.5) containing 0.5 µM atropine [16], [27]. Whole-cell membrane currents were recorded in response to application of agonists 5–7 days after RNA injection at a clamp potential of −70 mV. Currents were measured in response to application of various concentrations of agonists for 4 seconds. Between agonist applications, the recording chamber was washed with ND-96 buffer containing 0.5 µM atropine for 3 minutes. Responses were normalized to the maximum response induced by acetylcholine (ACh)(30 µM). The mean value of at least five oocytes was used for graphing concentration/response curves. Values are expressed ± standard error.

Data were analyzed with KaleidaGraph version 4.1 software for common statistical determinants (Synergy Software, Reading, PA). EC_50_, efficacy and Hill co-efficiency were obtained from the Hill equation as described [24].

## Results

### Design of (α6β2)_2_β3 AChR Constructs

**Figure 1:**
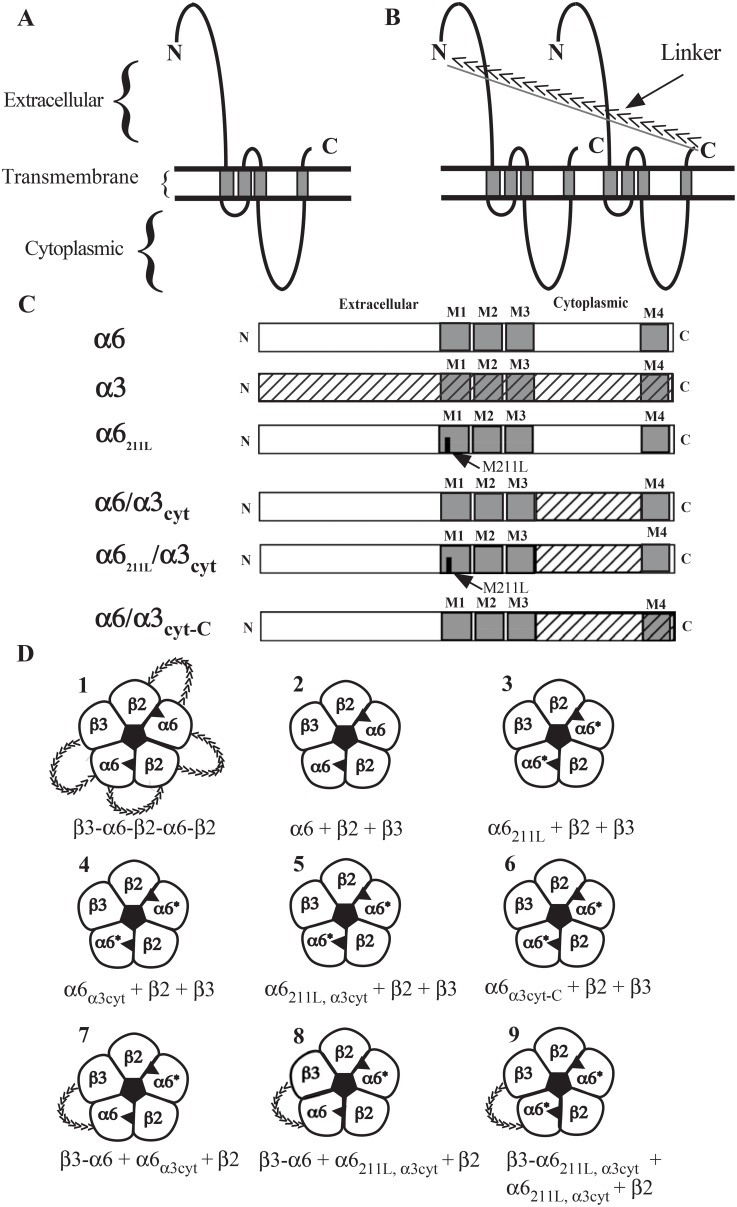
Illustration of (α6β2)_2_β3 AChR constructs. A) Diagrammatic representation of an AChR subunit. B) Diagrammatic representation of two AChR subunits joined by a linker. Direction of the linker is indicated by arrows. C) Representation of α6 and α3 sequences used in the α6/α3 chimeras studied. D) Representation of (α6β2)_2_β3 AChRs assembled from the various constructs. Agonist binding sites are shown as solid triangles between two subunits. The number and nomenclature for each construct depicted here are used in the following data figures.

Subunit concatamers and chimeras of α6 and α3 subunits were incorporated into a series of constructs for expressing (α6β2)_2_β3 AChRs as shown in Figure 1. Concatameric pentamer (construct **1**) linked through (AGS)_n_ linkers (Figure 1B and 1D) enabled successful expression of functional (α6β2)_2_β3 AChRs in oocytes [16]. Free native α6, β2, and β3 subunits (construct **2**) did not permit assembly of functional AChRs [12], [16]. To investigate the effect of exchanging methionine 211 and/or cytoplasmic domain of α6 subunit to that of α3, three chimeras were made to build constructs **3**, **4** and **5** (Figure 1C and 1D). Chimera α6_α3cyt-C_ and construct **6** were made to investigate the effect of the short C-terminus following the last transmembrane domain of the α6 subunit. To increase β3 incorporation, a QAP linker was used to link β3 with α6. Combining β3-QAP-α6 concatamer with various chimeras shown in Figure 1C, constructs **7**, **8** and **9** were made with one or two chimeric α6 subunits. Numbering and nomenclature of the constructs described above is used in the subsequent data figures.

### Expression Efficiency in Oocytes

**Figure 2:**
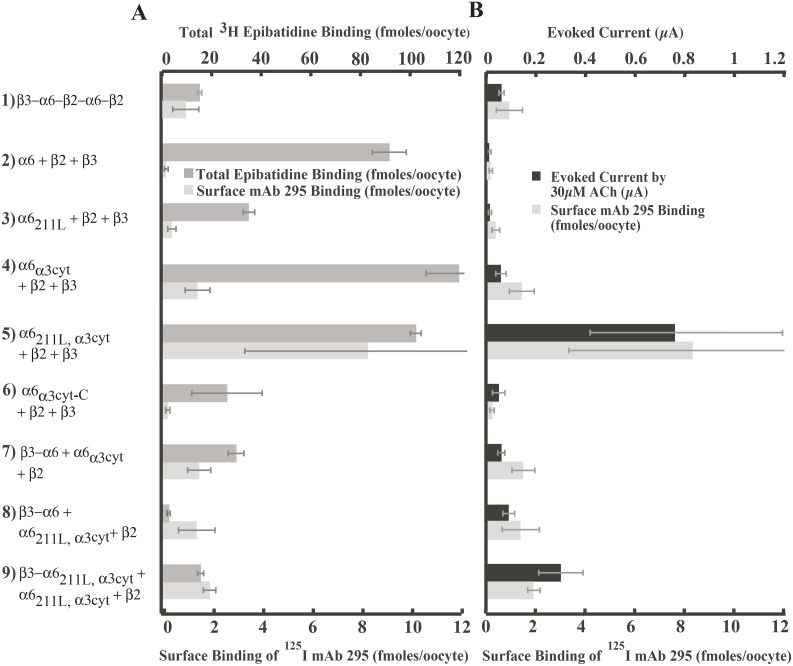
Efficiency of expression of functional (α6β2)_2_β3 AChRs using various constructs in *Xenopus* oocytes. The mRNAs of the nine constructs were injected on the same day and the assays were performed 6 days later. Values are the average of results from at least 8 oocytes. A) Comparison of total ACh binding sites versus surface expression for each (α6β2)_2_β3 AChR construct. Total number of ACh binding sites in partially or completly assembled AChRs inside cells plus in the surface membrane was assayed by binding of ^3^H epibatidine. B) Comparison of function versus surface expression for each (α6β2)_2_β3 AChR construct. Electrophysiological function was assayed by the current evoked by 30 µM in ACh voltage clamped oocytes. Mature AChRs on the oocyte surface were assayed by binding of ^125^I labeled mAb 295 to β2 subunits.

To evaluate efficiency of expression of the nine constructs in Figure 1, *Xenopus* oocytes were injected with mRNAs and six days later tested for total binding site assembly, surface protein level and responses to ACh. ACh binding sites were measured by binding of ^3^H epibatidine to immunoisolated detergent solubilized components containing β2 subunits. Expression in the surface membrane was assayed by binding to oocytes of ^125^I mAb 295 to β2 subunits. Biophysical properties were determined by examining the currents induced by 30 µM ACh. Results are shown in Figure 2.

Various constructs produced very different amounts of ACh binding sites (Figure 2A). The amounts of AChRs on the oocyte surface were not proportionate to the total amount of ACh binding sites. For example, constructs **2**, **4** and **5** all yielded more than 90 fmols of ^3^H epibatidine bound per oocyte but expressed very different amounts of AChRs on the surface, as low as 0.168±0.070 fmol for construct **2** or as high as 8.31±4.97 fmol for construct **5**. This indicates that some constructs result in incompletely assembled AChRs or properly assembled AChRs that were not transported to the cell surface, as shown previously [12], [16].

AChR function and AChR expression on the oocyte surface were closely correlated (Figure 2B), indicating that AChRs on the surface produced by most constructs had similar functional properties. The pentameric concatamer **1** (β3−α6−β2−α6−β2) exhibited the expected pharmacological properties and is considered the positive control [16]. Mature concatameric AChRs were assembled, but were inefficiently transported to the oocyte surface. Free native construct **2** (α6+β2+β3) subunits are the negative control. Free native subunits assembled large numbers of ACh binding sites. These α6β2* complexes assemble into amorphous aggregates, but virtually no mature AChRs [12]. Construct **3** (α6_211L_+β2+β3) did not increase surface expression or function, and decreased assembly of ACh binding sites. Construct **4** (α6_α3cyt_+β2+β3) greatly increased assembly with β2, surface expression, and function compared to construct **3**. Construct **5** (α6_211L,α3cyt_+β2+β3) combines the α3 components of constructs **3** and **4** in one α6 chimera. Construct **5** increased expression on the surface and function 40–80 fold compared to free subunits and 10–13 fold compared to concatamer **1**.

Other constructs did not approach the efficiency of expression obtained with construct **5** (α6_211L,α3cyt_+β2+β3). Construct **6** (α6_α3cyt-C_+β2+β3) reduced assembly of ACh binding sites, surface expression, and function compared to construct **4** (α6_α3cyt_+β2+β3). Thus, including α3 transmembrane domain 4 and C-terminal domain impaired assembly and transport. Subsequent experiments will show that pharmacological function was also altered. Construct **7** (β3−α6+α6_α3cyt_+β2) was intended to test whether the β3−α6 concatamer equaled or exceeded the effect of the α6_211L_ chimera in promoting assembly with β3 and whether a single α3 cytoplasmic domain was sufficient for enhanced assembly and transport. Construct **7** assembled fewer ACh binding sites than construct **4** with two α3 cytoplasmic domains, and was equally expressed on the surface. This is consistent with the ideas that assembly of β2 with α6 in the concatamers was reduced due to the absence of an α3 cytoplasmic domain in this α6 and that one α3 cytoplasmic domain per AChR is sufficient for transport to the surface. The β3−α6 concatamer provided no benefit. Construct **8** (β3−α6+α6_211L,α3cyt_+β2) had no better surface expression or function than **7** and lower assembly of ACh binding sites. Thus, the β3−α6 concatamer with a single or double α6 chimera provided no benefit. Construct **9** (β3−α6_211L,α3cyt_+α6_211L,α3cyt_+β2) expressed functional AChRs better than **8**. However, the β3−α6_211L,α3cyt_ concatamer substantially impaired assembly relative to that achieved with the α6 double chimeras as free subunits. This is consistent with the idea that this concatamer did not promote assembly of β3 with α6 and prevented M211 from promoting this assembly which does occur with free subunits. The β3−α6 concatamer used a QAP linker rather than the (AGS)_n_ linkers used in construct **1**. The change in linker may account for the ineffectiveness of the β3−α6 concatamer, but this linker has proven effective in several other constructs (unpublished).

**Figure 3:**
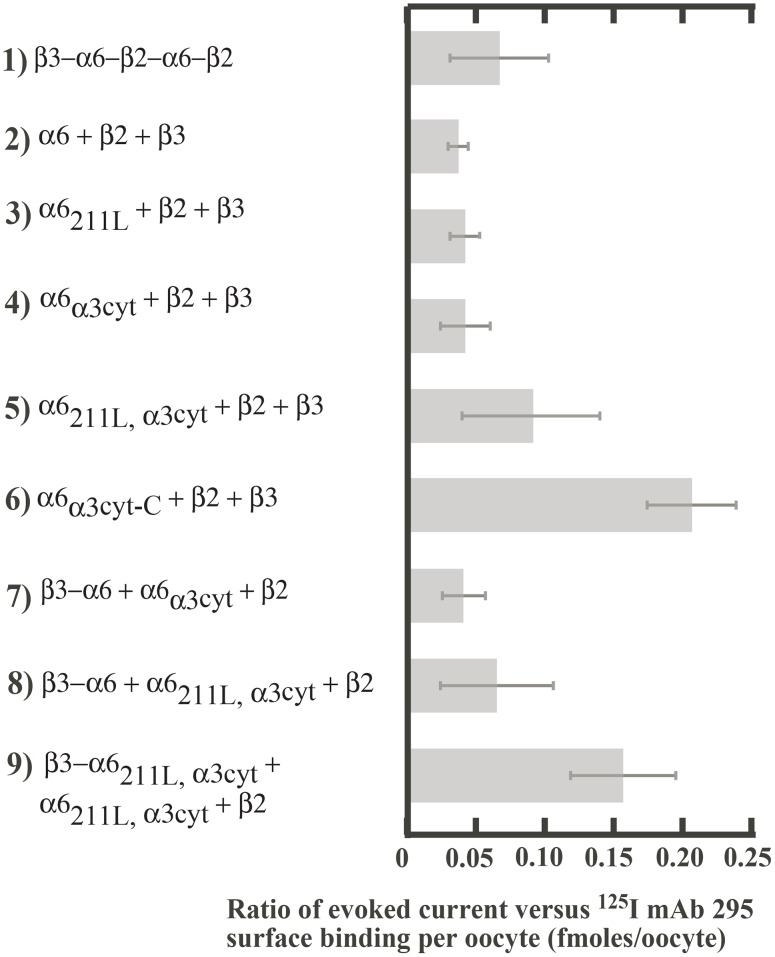
Effect of various constructs on channel properties. Evoked current was divided by the number of AChRs on the oocyte surface (i.e., mAb 295 surface binding) for each construct presented in Figure 2B. Values shown are a measure of effects on probability of channel opening and/or channel conductance.

Most constructs exhibited a similar ratio of current per surface AChR in response to activation by 30 µM ACh (Figure 3). This suggests that they have similar channel opening and/or channel conductance. Construct **6** (α6_α3cyt-C_+β2+β3) had larger current per surface AChR. This suggests that α3 transmembrane domain 4 and/or the extracellular C-terminal domain altered channel properties. Construct **9** also exhibited larger current per surface AChR for reasons which are not evident.

### Incorporation of β3 Accessory Subunit

**Figure 4:**
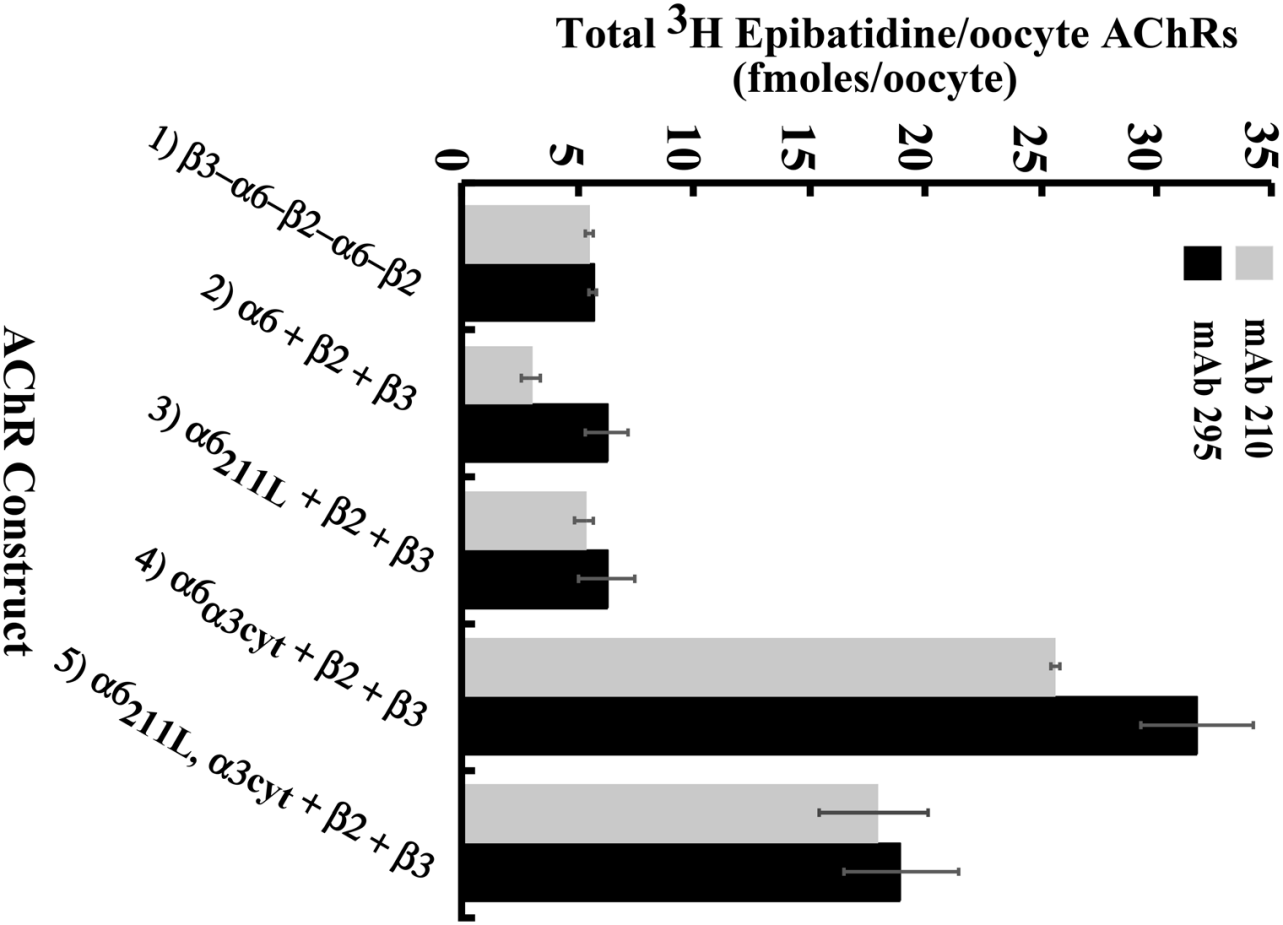
β3 incorporation in (α6β2)_2_β3 AChR constructs. Microwells were coated with mAb 295 to bind AChRs containing β2 or mAb 210 to bind AChRs containing β3. Detergent-solubilized AChRs were added to the wells. Bound AChRs were then assayed by binding of ^3^H epibatidine.

β3 functions only as an accessory subunit, thus does not form ACh binding sites with α subunits. As expected, incorporation of β3 is complete in concatamers of construct **1** (β3−α6−β2−α6−β2, Figure 4). Incorporation of β3 is least in the large number of partially assembled and aggregated α6β2 ACh binding site complexes formed from the free native subunits of construct **2** (α6+β2+β3, Figure 4). Changing only the α6 methionine 211 to leucine in construct **3** (α6_211L_+β2+β3) results in complete incorporation of β3 (Figure 4) and formation of functional AChRs (Figure 2B). Thus, this part of α6 transmembrane domain 1 on the side of α6 where the accessory subunit is expected to assemble [19], contributes to assembly of β3. Changing only the large cytoplasmic domain of α6 for that of α3 in construct **4** (α6_α3cyt_+β2+β3) increases assembly of β3, but not as effectively as changing the single amino acid M211 (Figure 4). However, the α3 cytoplasmic domain is efficient at increasing both assembly with β2 and transport to the cell surface (Figure 2A and Figure 4). Combination of the two chimeras in construct **5** exhibited complete incorporation of β3 (Figure 4) and efficient assembly of mature AChRs contributed by M211L (Figure 2B) with the efficient assembly with β2 and transport to the surface contributed by the cytoplasmic domain of α3 (Figure 2A) to produce large amounts of functional AChRs on the cell surface.

### Efficiency of Mature (α6β2)_2_β3 AChR Assembly

**Figure 5:**
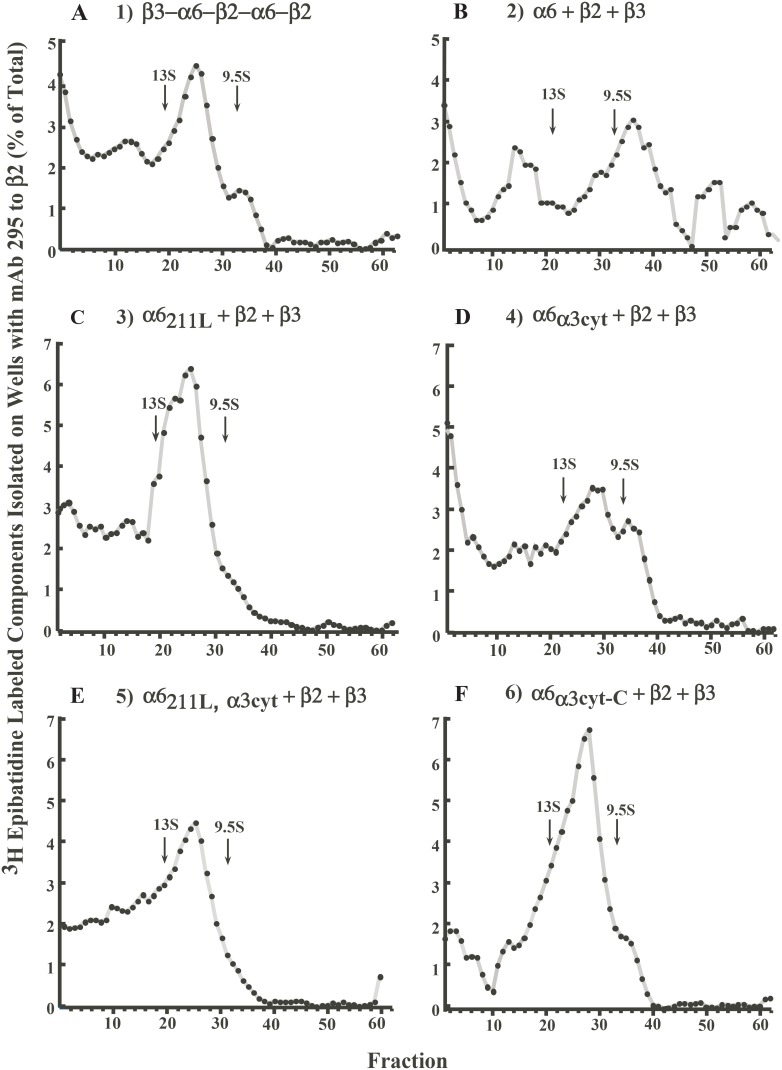
Assembly of (α6β2)_2_β3 AChRs constructs evaluated by sucrose sedimentation velocity gradient analysis. After centrifugation, gradient fractions were immunoisolated on microwells coated with mAb 295 to β2 to isolate α6β2β3 AChRs prior to labeling with ^3^H epibatidine. Properly assembled mature (α6β2)_2_β3 AChRs sediment between the two internal standards, 9S monomer and 13S dimer of *Torpedo californica* AChRs. Peaks on the left of dimers in the gradient indicate multimers or aggregates of AChRs, while peaks on the right of monomers represent partially assembled AChRs. A) Expression of pentameric concatamer construct **1** (β3−α6−β2−α6−β2) resulted in a high proportion of mature AChRs, as expected (16). B) Expression of construct **2** (α6+β2+β3) resulted in a high proportion of aggregates and partially assembled AChRs and a very low proportion of mature AChRs, as expected (12). C) Expression of construct **3** (α6_211L_+β2+β3) resulted in a large proportion of mature AChRs. D) Expression of construct **4** (α6_α3cyt_+β2+β3) resulted in mature AChRs but also partially assembled AChRs and both large and very large aggregates. E) Expression of construct **5** (α6_211L,α3cyt_+β2+β3) showed mature AChRs and some aggregates. F) Expresion of construct **6** (α6_α3cyt-C_+β2+β3) showed a high proportion of mature AChRs and few aggregates.

Efficiency of assembly of mature AChR was analyzed by sedimentation velocity analysis on sucrose gradients (Figure 5). Confirming previous observations [16], the pentameric concatamer construct **1** exhibited a high proportion of mature AChRs of the expected size, intermediate between monomers and dimers of Torpedo AChRs. Construct **2** (α6+β2+β3) resulted in high proportions of partially assembled AChRs and large aggregates, but virtually no mature AChRs. This confirms previous observations that free wild type subunits do not assemble into mature AChRs in *Xenopus* oocytes [12]. Construct **3** (α6_211L_+β2+β3) resulted in a substantial proportion of mature AChRs. Thus, replacing the methionine unique to α6 in transmembrane domain 1 with the leucine found in α3 and most other α subunits greatly promoted assembly of mature AChRs. Construct **4** (α6_α3cyt_+β2+β3) resulted in mature AChRs, but also some partially assembled AChRs and a substantial proportion of large aggregates. Figure 2 showed that the absolute amounts of ACh binding sites assembled, AChRs transported to the surface, and functional AChRs were greater with the α6 chimera incorporating only the large cytoplasmic domain of α3 (construct **4**) than with the chimera incorporating only leucine 211 of α3 (construct **3**). Combining the two chimeras in an α6 subunit in construct **5** (α6_211L,α3cyt_+β2+β3) gave a synergistic effect. The proportion of mature AChRs was large, and partially assembled AChRs and the largest aggregates were eliminated (Figure 5E). In addition, the absolute amount of AChRs assembled and transported to the surface membrane where their function was assayed increased 13–80 fold compared to when the chimeras were expressed individually (Figure 2). Construct **6** (α6_α3cyt-C_+β2+β3) produced a high proportion of mature AChRs (Figure 5F). Thus, the α3 transmembrane domain 4 and/or the α3 C-terminal extracellular domain contributed to assembly of an increased proportion of mature AChRs and a decreased proportion of large aggregates and assembly intermediates compared to the α3 cytoplasmic domain alone (Figure 5D). However, the absolute amount of functional AChRs on the cell surface was lower than with only the α3 cytoplasmic domain in construct **4** (Figure 2B). Constructs containing β3−α6 concatamers in constructs **7**, **8**, and **9** exhibited substantial proportions of mature AChR (Figure S1), although the absolute amounts of AChRs assembled or functional AChRs on the cell surface were much smaller than achieved with construct **5** containing the α6 double chimera, β2, and β3 as free subunits.

### Pharmacology of (α6β2)_2_β3 AChR Constructs

**Figure 6:**
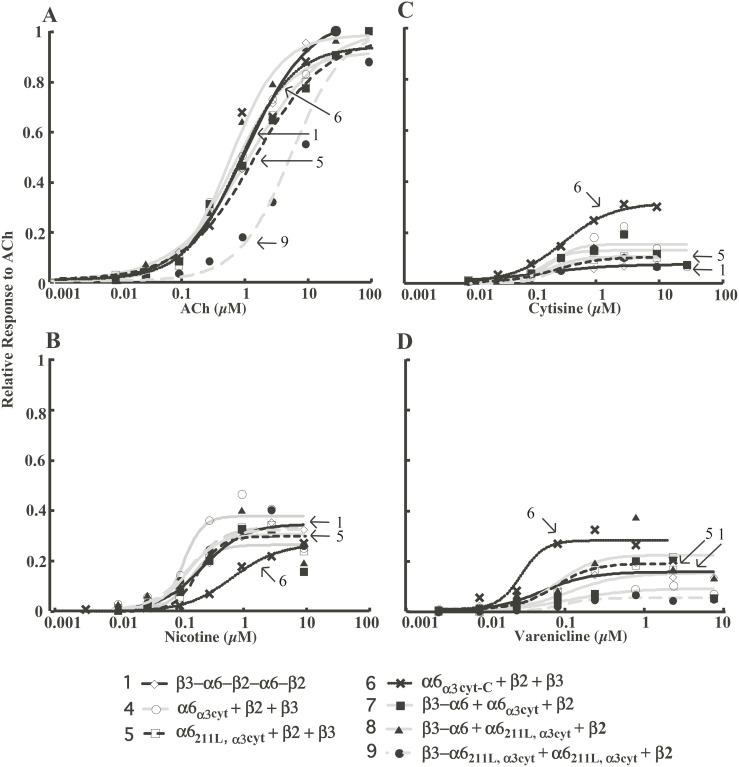
Concentration/response curves for constructs that resulted in significant amounts of functional AChRs. Full agonist (ACh) and partial agonists (cytisine, nicotine, and varenicline) were used on (α6β2)_2_β3 AChRs. Each point is the average response of at least 5 oocytes. Arrows indicate construct **5**, that behaves like construct **1**, and constructs **6** and **9** that are divergent.

**Figure 7:**
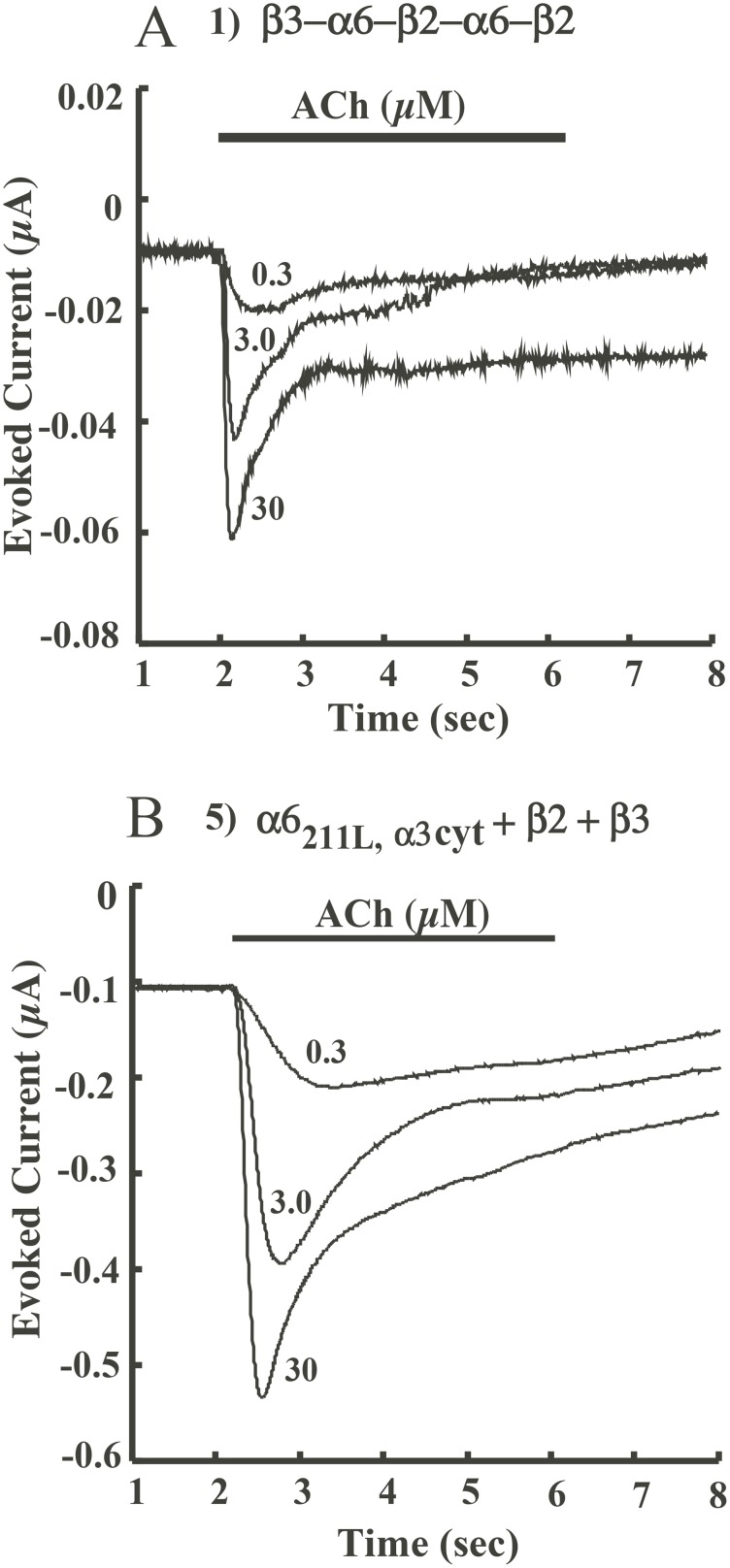
Kinetics of responses to increasing concentrations of ACh by constructs 1 and 5. A) Concatamer **1** (β3−α6−β2−α6−β2) responds more rapidly with greater currents and more extensive desensitization at higher ACh concentrations. B) Construct **5** (α6_211L,α3cyt_+β2+β3) response kinetics to ACh are similar, but amplitudes of responses are much larger.

Although all of the (α6β2)_2_β3 AChR constructs retain the α6 extracellular domain for agonist binding (Figure 1), altering transmembrane and cytoplasmic domains may change the pharmacology of (α6β2)_2_β3 AChRs. Therefore, acetylcholine and various full and partial agonists of AChRs were used to investigate activation and desensitization of (α6β2)_2_β3 AChR constructs (Figure 6, 7).

Most constructs exhibited similar high sensitivities to activation by agonists (Figure 6, Table 1). Constructs **2** and **3** showed very little activation by acetylcholine, with maximum responses less than 20 nA. Thus their pharmacology was not studied in detail. EC_50_ of acetylcholine for activating all constructs, except **9**, is around 1 µM. Kinetics of activation by ACh are displayed in Figure 7 and Figure S2. Constructs exhibited similar kinetics of activation and desensitization by various concentrations of ACh. For example, construct **5** (α6_211L,α3cyt_+β2+β3) responded to 30 µM ACh with 10 fold greater amplitude than construct **1** (β3−α6−β2−α6−β2) but had similar response kinetics. There were noticeable concentration-dependent shifts in peak times in constructs **5**, **6** and **7** (Figure 7 and Figure S2). The response persists after the ACh application period due to design of our electrophysiology testing apparatus, permitting washout to be slower than wash in. This effect might also result from calcium-activated chloride channels that remain open longer than the AChRs channels. Theoretically this effect could be avoided by omitting Ca^++^ from outside solution or clamping at a lower voltage. However, Ca^++^ may potentiate activation of AChRs by agonists and α6* AChR evoked peak current is strongly influenced by extracellular Ca^++^ [28], [29]. Considering the overall low functional responses of the nine constructs, experiments were all executed at a holding potential of −70 mV and in a buffer with normal concentration of Ca^++^. Finally, altered kinetics of agonist responses were only exhibited by constructs that were not especially effective, thus characterizing them in detail is tangential to our goal of effectively expressing (α6β2)_2_β3 AChRs.

**Table 1:** Pharmacological Properties of Various (α6β2)_2_β3 AChR Constructs.

	AChR Construct						
**Agonist**	**1**	**4**	**5**	**6**	**7**	**8**	**9**
	β3−α6−β2−α6−β2	α6_α3cyt_+β2+β3	α6_211L,α3cyt_+β2+β3	α6_α3cyt-C_+β2+β3	β3**–**α6+α6_α3cyt_+β2	β3**–**α6+α6_211L,α3cyt_+β2	β3**–**α6_211L,α3cyt_+α6_211L,α3cyt_+β2
**ACh**							
EC_50_ (µM)	1.21±0.16	0.740±0.096	0.33±0.25	0.756±0.262	1.40±0.348	0.669±0.067	6.06±2.68
**Nicotine**							
EC_50_ (µM) Efficacy (%)	0.239±0.031 34.5±2.4	0.116±0.044 37.8±4.9	0.203±0.054 30.7±26.7	0.711±0.120 27.5±1.6	0.155±0.081 26.2±5.0	0.135±0.083 30.5±5.4	0.145±0.055 33.3±3.5
**Cytisine**							
EC_50_ (µM) Efficacy (%)	0.189±0.052 17.0±1.0	0.219±0.152 14.9±2.9	0.256±0.020 10.4±0.2	0.336±0.047 32.3±1.3	0.160±0.102 12.5±2.1	0.198±0.139 9.92±2.10	0.221±0.146 9.46±1.99
**Varenicline**							
EC_50_ (µM) Efficacy (%)	0.0620±0.0248 16.1±1.5	0.138±0.071 9.37±1.18	0.104±0.012 18.3±0.7	0.0374±0.0137 27.6±3.0	0.138±0.166 15.6±4.9	0.0978±0.0951 22.9±6.1	0.114±0.036 5.59±0.06

Note the EC_50_ values for construct **6**, which contains the α3 cytoplasmic domain, transmembrane domain 4, and the extracellular C-terminal domain, are significantly different from those of construct **1** for nicotine (p = 0.0266).

Partial agonists nicotine, cytisine, and varenicline activate constructs similarly, except construct **6** (Figure 6 and Table 1). Construct **6** (α6_α3cyt-C_+β2+β3) is an outlier in the concentration/response curves for nicotine, cytisine, and varenicline (Figure 6) and EC_50_ values for nicotine and varenicline (Table 1). This suggests that α3 transmembrane domain 4 and/or the C-terminal domain alter sensitivity to activation.

Besides difference in activation, construct **6** (α6_α3cyt-C_+β2+β3) also exhibits more rapid desensitization than the other constructs (Table 2 and Figure S3). The desensitization rate of construct **6** increased 48% compared to that of construct **1**. Construct **5,** which has both M211L and the α3 large cytoplasmic domain, desensitized at a rate close to construct **1** (Table 2). Thus, α3 transmembrane domain 4 and/or the α3 C-terminal extracellular domain increase the rate of desensitization.

**Table 2:** T_1/2_ of Various (α6β2)_2_β3 AChR Constructs against 30µM ACh.

	AChR Construct						
	**1**	**4**	**5**	**6**	**7**	**8**	**9**
	β3−α6−β2−α6−β2	α6_α3cyt_+β2+β3	α6_211L,α3cyt_+β2+β3	α6_α3cyt-C_+β2+β3	β3**–**α6+α6_α3cyt_+β2	β3**–**α6+α6_211L,α3cyt_+β2	β3**–**α6_211L,α3cyt_+α6_211L,α3cyt_+β2
**T_1/2_**	0.572±0.061	0.535±0.064	0.654±0.127	0.302±0.086	0.580±0.044	0.105±0.038***	0.554±0.033

## Discussion

It is hard to study a single AChR subtype *in vivo* because various subtypes often co-express together in brain. Establishing *in vitro* model systems expressing α6-containing AChRs is challenging. By changing a single unique amino acid in transmembrane domain 1 of α6 and the cytoplasmic domain of α6 to that of α3, we succeeded in efficiently expressing in *Xenopus* oocytes large amounts of human (α6β2)_2_β3 AChRs. This will be very useful for characterizing functional properties of these AChRs and drugs directed at them.

These AChRs have the pharmacological properties of (α6β2)_2_β3 AChRs formed from wild type subunits linked in a concatamer. As discussed in our previous study, concatameric (α6β2)_2_β3 AChRs showed the pharmacology expected from measuring dopamine release in the brain tissue [16]. EC_50_ values for nicotine obtained *in vivo* ranged from 0.1 µM to 1 µM for activation of α6* AChRs not containing α4 [5], [21], [30]. Such variation may be due to technical variations. The pentameric concatamer and AChRs expressed from the free subunits of our highest expressing free subunit construct **5** have very similar pharmacological properties, (e.g. EC_50_ for nicotine = 0.239 or 0.203 µM) but most of the constructs exhibit similar properties.

Expression from these modified free subunits is much more efficient than expression from (α6β2)_2_β3 concatamers [16]. Concatamers will still be required to ensure efficient expression and subunit order of the more complex (α6β2)(α4β2)β3 AChR subtype [16]. Dopaminergic neurons which uniquely express α6β2β3* AChRs may have chaperones for efficiently assembling β3 accessory subunits with α6 and ensuring efficient assembly of (α6β2) ACh binding sites in AChRs with (α4β2) ACh binding sites [12], [13]. In cells expressing free α4, α6, and β2 subunits, α6 and β2 do not effectively assemble to form (α6β2)(α4β2)* AChRs, instead α4β2 AChRs predominate (Kuryatov unpublished).

Chimeras of α6 subunits with α3 subunits have revealed sequences of α6 that inhibit expression of α3β2 AChRs and sequences of α3 that permit expression of α6β2 AChRs [12], [16]. Studies reported here build on this foundation to achieve efficient expression of (α6β2)_2_β3 AChRs and further explain structural limits to α6* AChR expression.

The unique methionine 211 in the first transmembrane domain of α6, when exchanged for the leucine present in most other α subunits, promotes efficient assembly with β3 and formation of a high proportion of mature AChRs. This suggests that a unique chaperone in dopaminergic neurons might bind to this region of α6 to promote assembly with β3. No chaperone is needed to get efficient assembly of β3 with α4 subunits that have a leucine at this position [22]. Replacing leucine 211 of α3 with methionine inhibits assembly of α3β2 AChRs [16].

The concatameric construct **1** (β3−α6−β2−α6−β2) efficiently assembles into mature AChRs, but these are not efficiently transported to the cell surface, as are the concatamers β3−α6−β2−α4−β2 or β3−α4−β2−α6−β2 [16]. This suggests that the large cytoplasmic domain of α4 may contribute to transport to the cell surface. The cytoplasmic domain of α4 is uniquely large. Here we show the normal sized cytoplasmic domain of α3, a subunit closely related to α6 in sequence, when substituted for that of α6 can promote both transport to the cell surface and increase assembly with β2 subunits. It is known that large cytoplasmic domain chimeras can increase the assembly and transport of AChRs [17]. Incorporation of shorter sequences of α3 cytoplasmic domain will be required to determine the minimum sequences required to promote assembly with β2 or surface transport. Use of shorter α3 cytoplasmic domain sequences might avoid incorporation of the α3 amphipathic α helix which could alter cation selectivity [17], [31]. Identification of these shorter sequences may further suggest their mechanisms of action in transport and assembly and help to explain their collaboration with the single amino acid change M211L to promote expression of human (α6β2)_2_β3 AChRs from free subunits in *Xenopus* oocytes.

The synergism of these two chimeras appears to result from 211L promoting a high proportion (but low amount) of assembly of β3 combined with the cytoplasmic domain of α3 promoting extensive assembly with β2, and to a lesser extent β3, and facilitating transport of assembled AChRs to the oocyte surface. Linking β3 to α6 in a concatamer can force their assembly [16]. However, the dimeric concatamer used here did not exceed the effect of combining 211L and α3 cytoplasmic domain in generating functional (α6β2)_2_β3 AChRs.

The α6 chimeras with 211L and the α3 large cytoplasmic domain (construct **5**) effectively expressed functional (α6β2)_2_β3 AChRs without altering their pharmacological properties. Transfection of HEK 293 cells with a 2∶1∶10 ratio of a chimera with the extracellular domain of α6 and the rest of α3, β2, and β3 with and a V9’S gain of function mutation resulted in functional (α6β2)_2_β3 AChRs [32]. Despite the extensive modifications necessary in those studies to assemble β3 and get α6 to the surface of the cell line, some of the pharmacological properties of its AChRs were similar to constructs **1** and **5**, while others were divergent. On the cell line, EC_50_ values for ACh (0.23 µM), nicotine (0.15 µM), cytisine (0.072 µM), and varenicline (0.022 µM) were lower than we observed by 1.4 to 5.9 fold, as might be expected as a result of their using a hyperactive mutant β3 subunit. Similarly, the efficacy for nicotine they observed was two fold greater than we observed for constructs **1** and **5**.

In conclusion, we simplified and improved expression of (α6β2)_2_β3 AChRs in *Xenopus* oocytes while maintaining their pharmacological properties. The critical structural features that we identified as important for assembly and transport to the surface will be beneficial for understanding what happens *in vivo* and in developing α6* cell lines for drug screening.

## Supporting Information

Figure S1 **Sucrose sedimentation velocity gradient analysis of the size of ^3^H epibatidine binding components formed by various constructs.** Monomers (9.5S) and dimers (13S) of *Torpedo californica* AChR were sedimented as internal standards. A) Expression of construct **7** (β3−α6+α6_α3cyt_+β2) resulted in some very large aggregates, a substantial proportion of mature AChRs, and some partially assembled AChRs. B) Expression of construct **8** (β3−α6+α6_211L,α3cyt_+β2) resulted in aggregates, a substantial proportion of mature AChRs, and significant amounts of partially assembled AChRs. C) Expression of construct **9** (β3−α6 α6_211L,α3cyt_+α6_211L,α3cyt_+β2) resulted in a high proportion mature AChRs, and a substantial fraction of partially assembled AChRs.
(TIF)

Figure S2 **Kinetics of responses to increasing concentrations of ACh by constructs 4, 6, 7, 8 and 9.** A) Construct **4** (α6_α3cyt_+β2+β3). B) Construct **6** (α6_α3cyt-C_+β2+β3). C) Construct **7** (β3−α6+α6_α3cyt_+β2). D) Construct **8** (β3−α6_211L_+α6_α3cyt_+β2). E) Construct **9** (β3−α6_211L,α3cyt_+α6_211L,α3cyt_+β2).
(TIF)

Figure S3 **Response kinetics to 30 µM ACh by constructs 1, 4–9.**
(TIF)

Figure S4 **Stability of concatamer in construct 8 (β3**−**α6+α6_211L_,_α3cyt_+β2) was confirmed by western blot.** AChR solubilized from 100 oocytes solubilized by Triton X-100 was purified and concentrated by immunoaffinity chromatography using mAb 295 linked to resin before being resolved to subunits by SDS-polyacrylamide gel electrophoresis. After overnight incubation at 4°C, the column was spun at 5,000 rpm for 15 minutes to remove unbound material and washed with PBS, 0.5% Triton solution. 40 µl of LDS sample buffer (Invitrogen) was placed in the column and heated for 30 minutes at 37°C. The eluent was resolved by SDS-polyacrylamide gel electrophoresis and then transferred using a semidry electroblotting method [16]. Blots were then quenched with 5% Carnation dried nonfat milk for in PBS, 0.5% Triton X-100, 10 mM NaN_3_ for one hour. Blots were probed with rat antiserum to α6 (1∶500) [13], then incubated with 2 nM ^125^I-labeled goat anti-rat IgG for 3 h at room temperature. After washing in 0.5% Triton with NaN_3_, blots were visualized by autoradiography. Proteins of the sizes expected of QAP linker β3−α6 (∼8.3×10^4^ Da) and corresponding free subunit α6_211L_,_α3cyt_ (∼5×10^4^ Da) were obtained without signs of proteolytic degradation.
(TIF)
