# Supplementary figures and images for: Efficient Expression of Functional (α6β2)2β3 AChRs in Xenopus Oocytes from Free Subunits Using Slightly Modified α6 Subunits
Source: PLoS One. 2014 Jul 28;9(7):e103244. doi: 10.1371/journal.pone.0103244 (PMC4113361; doi:10.1371/journal.pone.0103244)

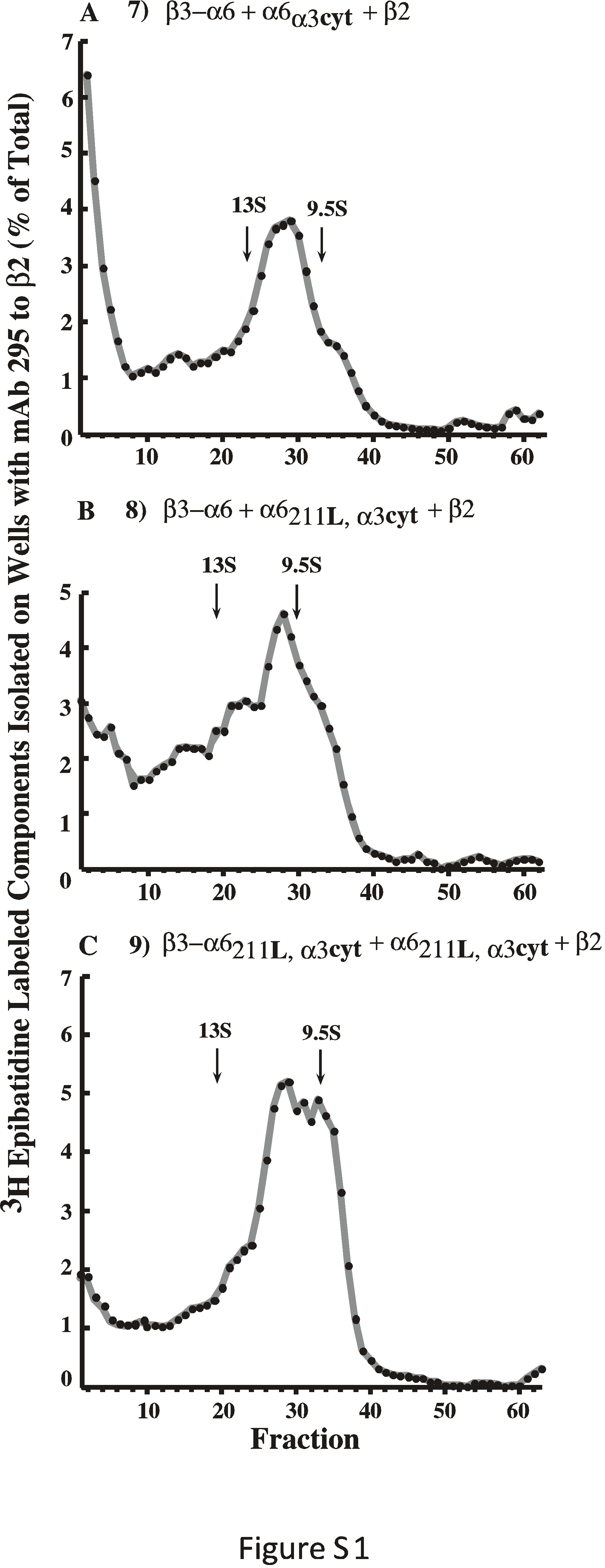

Supplement: Figure S1 — Sucrose sedimentation velocity gradient analysis of the size of 3H epibatidine binding components formed by various constructs. Monomers (9.5S) and dimers (13S) of Torpedo californica AChR were sedimented as internal standards. A) Expression of construct 7 (β3−α6+α6α3cyt+β2) resulted in some very large aggregates, a substantial proportion of mature AChRs, and some partially assembled AChRs. B) Expression of construct 8 (β3−α6+α6211L,α3cyt+β2) resulted in aggregates, a substantial proportion of mature AChRs, and significant amounts of partially assembled AChRs. C) Expression of construct 9 (β3−α6 α6211L,α3cyt+α6211L,α3cyt+β2) resulted in a high proportion mature AChRs, and a substantial fraction of partially assembled AChRs. (TIF) [file pone.0103244.s001.tif]

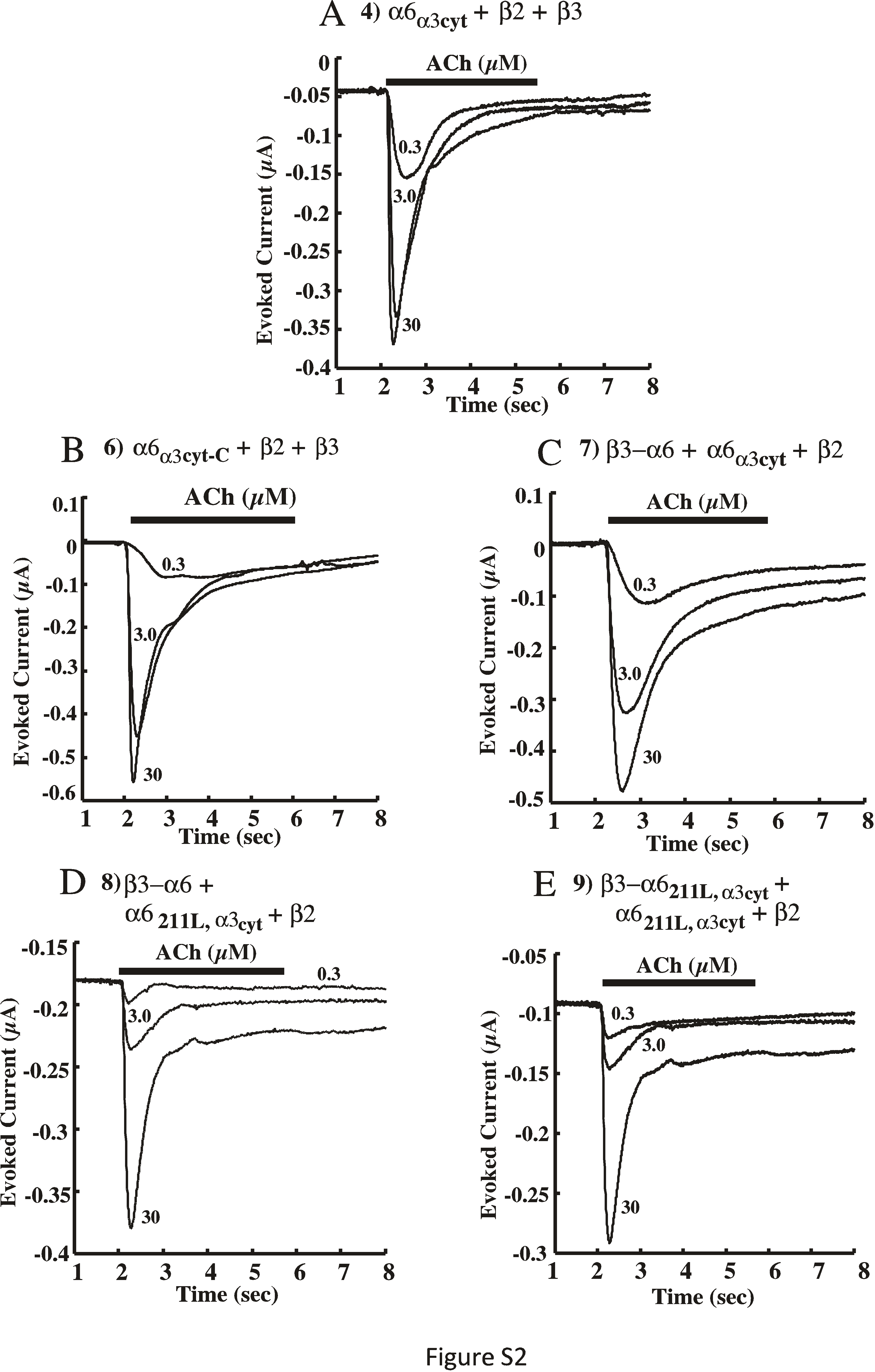

Supplement: Figure S2 — Kinetics of responses to increasing concentrations of ACh by constructs 4, 6, 7, 8 and 9. A) Construct 4 (α6α3cyt+β2+β3). B) Construct 6 (α6α3cyt-C+β2+β3). C) Construct 7 (β3−α6+α6α3cyt+β2). D) Construct 8 (β3−α6211L+α6α3cyt+β2). E) Construct 9 (β3−α6211L,α3cyt+α6211L,α3cyt+β2). (TIF) [file pone.0103244.s002.tif]

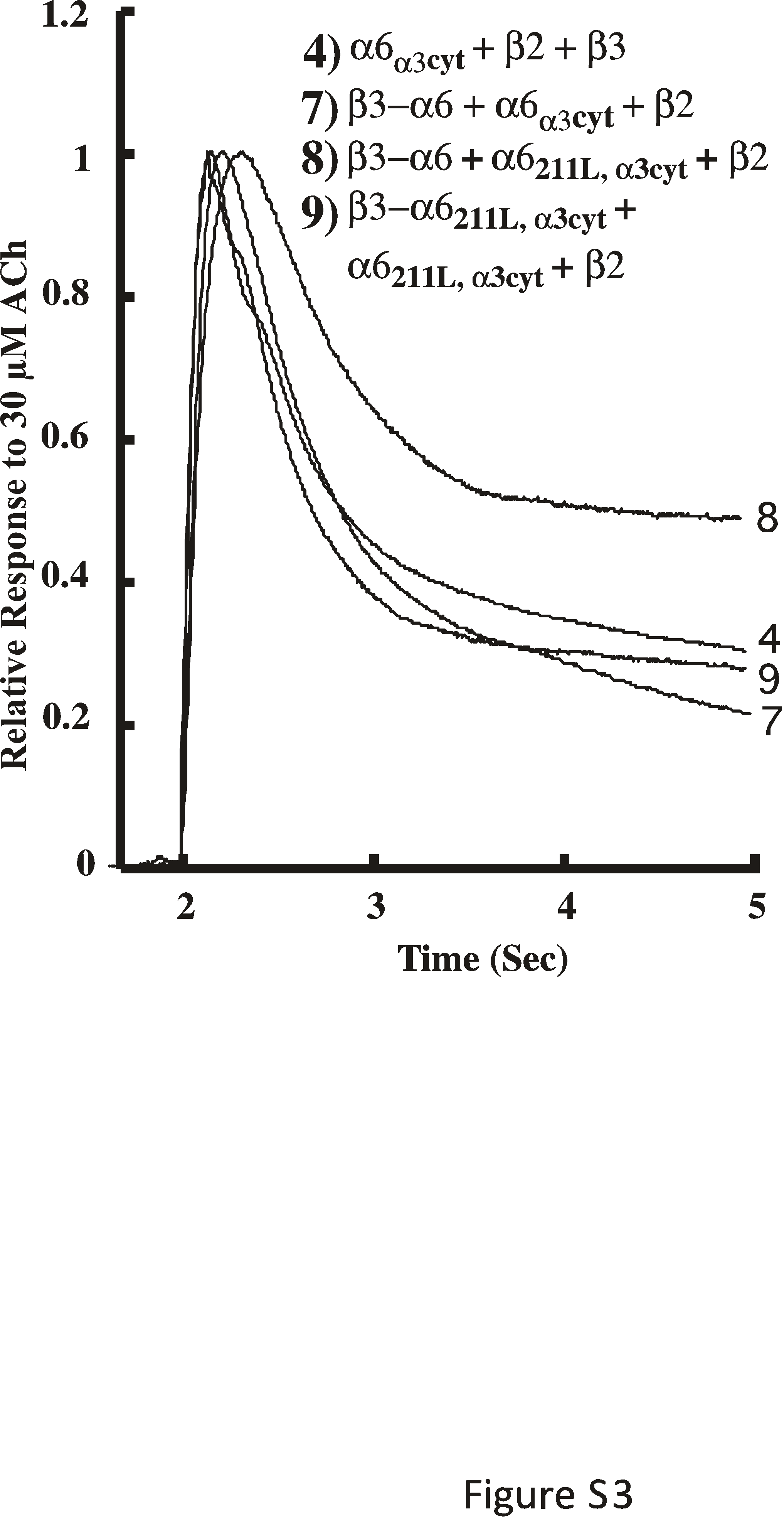

Supplement: Figure S3 — Response kinetics to 30 µM ACh by constructs 1, 4–9. (TIF) [file pone.0103244.s003.tif]

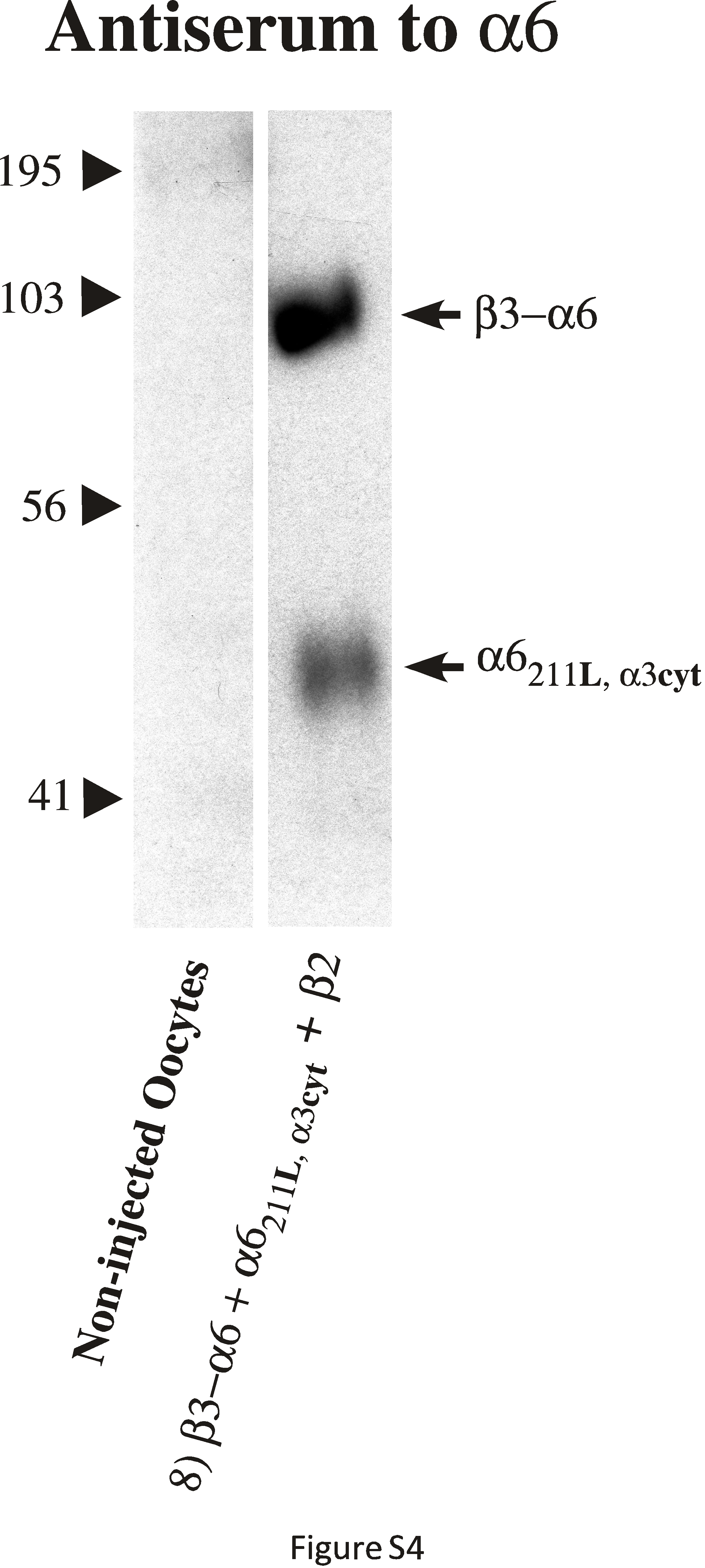

Supplement: Figure S4 — Stability of concatamer in construct 8 (β3−α6+α6211L,α3cyt+β2) was confirmed by western blot. AChR solubilized from 100 oocytes solubilized by Triton X-100 was purified and concentrated by immunoaffinity chromatography using mAb 295 linked to resin before being resolved to subunits by SDS-polyacrylamide gel electrophoresis. After overnight incubation at 4°C, the column was spun at 5,000 rpm for 15 minutes to remove unbound material and washed with PBS, 0.5% Triton solution. 40 µl of LDS sample buffer (Invitrogen) was placed in the column and heated for 30 minutes at 37°C. The eluent was resolved by SDS-polyacrylamide gel electrophoresis and then transferred using a semidry electroblotting method [16]. Blots were then quenched with 5% Carnation dried nonfat milk for in PBS, 0.5% Triton X-100, 10 mM NaN3 for one hour. Blots were probed with rat antiserum to α6 (1∶500) [13], then incubated with 2 nM 125I-labeled goat anti-rat IgG for 3 h at room temperature. After washing in 0.5% Triton with NaN3, blots were visualized by autoradiography. Proteins of the sizes expected of QAP linker β3−α6 (∼8.3×10^4^ Da) and corresponding free subunit α6211L,α3cyt (∼5×10^4^ Da) were obtained without signs of proteolytic degradation. (TIF) [file pone.0103244.s004.tif]
